# Body image and social media as predictors of pregnancy health behaviors

**DOI:** 10.1038/s41598-026-43123-5

**Published:** 2026-03-05

**Authors:** Agnieszka Bień, Grażyna Bączek, Beata Pięta, Bożena Kulesza-Brończyk, Dorota Ćwiek, Joanna Grzesik-Gąsior

**Affiliations:** 1 Chair of Obstetrics Development, Faculty of Health Sciences, Medical University of Lublin, Lublin, Poland; 2 Department of Obstetrics and Gynecology Didactics, Medical University of Warsaw, Warsaw, Poland; 3 Department of Practical Midwifery Education, Poznan University of Medical Sciences, Poznan, Poland; 4 Department of Obstetrics, Gynaecology and Maternity Care, Faculty of Health Sciences, Medical University of Bialystok, Bialystok, Poland; 5 Department of Obstetrics and Pathology of Pregnancy, Faculty of Health Sciences, Pomeranian Medical University in Szczecin, Szczecin, Poland; 6 State University of Applied Sciences in Krosno, Krosno, Poland

**Keywords:** Pregnancy, Health behaviors, Body image, Social media, Prenatal education, Health care, Psychology, Psychology, Risk factors

## Abstract

Pregnancy is a time of profound physical and psychological change, during which health behaviors can affect maternal and fetal outcomes. This study aimed to examine how body image and perceived media portrayals of pregnancy are associated with health behaviors among pregnant women. A cross-sectional survey was conducted with 921 participants across multiple Polish regions. Standardized instruments measured health-promoting behaviors, body esteem, and perceptions of media portrayals of pregnancy. Hierarchical regression analysis was used to identify clinical, psychological, and sociocultural predictors. Results showed that advancing gestational age was associated with a decline in health behaviors (B = -0.161; *p* < 0.001), while comorbidities predicted an increase (B = 2.871; *p* < 0.001). Negative media portrayals were linked to lower engagement (B = -2.938; *p* < 0.001), whereas positive portrayals and media underrepresentation correlated with increased health practices. Components of body esteem, especially physical condition (B = 0.812; *p* < 0.001), were the strongest predictors. The final model explained 87% of the variance in health behaviors (R² = 0.87; F = 433.54; *p* < 0.001). These findings underscore the need for holistic prenatal health promotion that incorporates body image, media perceptions, and psychosocial, clinical, and sociocultural factors to improve education and support outcomes.

**Supplementary Information:**

The online version contains supplementary material available at 10.1038/s41598-026-43123-5.

## Introduction

Pregnancy is a period of intense physiological, psychological, and social changes, and requires appropriate care to support the health of the pregnant person and healthy fetal development. In this context, health behaviors play a significant role in shaping well-being during pregnancy and can directly affect maternal and fetal outcomes. Both health-promoting and health-risk behaviors can drastically modify the course and outcomes of pregnancy^1,2^. A balanced diet, regular physical activity, rest, avoiding harmful substances, and adhering to medical recommendations are examples of health-promoting behaviors, whereas health-risk behaviors include smoking, unhealthy alcohol use, an unhealthy diet, and physical inactivity^3,4^. Risky behaviors are linked to an increased risk of obstetric complications, premature delivery, or low birth weight. Understanding the mechanisms underlying these choices is therefore essential to effectively support people during pregnancy in making decisions that promote their own and the baby’s health^5^.

The perception of one’s own body is one of the important psychosocial areas influencing these choices. The body changes dynamically during pregnancy, which can affect self-esteem, psychological well-being, and social relationships. Body image, understood as subjective perceptions and emotional evaluations of one’s own appearance, can both support healthy choices and contribute to anxiety, pressure, and compensatory behaviors. During pregnancy, body image is a particularly sensitive psychological domain, shaped by rapid and substantial physical changes^6,7^.

With the development of information technology, the role of social media as a source of influence on health behavior is also growing. One important aspect of this influence is the formation of body image and expectations of motherhood. Nowadays, social media serves not only as an information source, but also as a platform for forming opinions. Many people during pregnancy rely on them for information, emotional support, and peer-shared experiences and advice^8,9^. It is estimated that as many as 97.5% of pregnant women use the internet to search for information on pregnancy, newborn care and the postpartum period, and 89% actively contribute on social media platforms such as Facebook, Instagram, and TikTok^10^. In addition to educational content, these media frequently promote unrealistic portrayals of pregnancy (e.g., presenting pregnant women as well-groomed, happy, and attractive), which may foster appearance-related social comparison and body dissatisfaction, as suggested in the broader body image literature and increasingly discussed in perinatal contexts^11,12^.

The lack of content moderation and verification of advice on parenting forums or by influencers increases the risk of misinformation and the promotion of recommendations that contradict current medical knowledge. The pressure to create an unrealistic image, along with changes in the body, can lead to negative perceptions about one’s own body and pregnancy. Body dysmorphic disorder (BDD) has been associated with low self-esteem and lower psychological well-being^13,14^. As a result, it can affect health decisions made during pregnancy. Women with a history of eating disorders or with low body self-esteem are particularly vulnerable to adaptation difficulties^6^.

Previous studies indicate that a complex interaction of biological, psychological and environmental factors shapes health behaviors during pregnancy. However, few studies have simultaneously examined the influence of social media and psychosocial mechanisms related to body perception during pregnancy. In an era of intense digitization of health and rising online activity during pregnancy, integrating these domains may be important for understanding current health patterns. From a theoretical perspective, these associations can be understood within sociocultural frameworks, particularly the Tripartite Influence Model (TIM). The TIM proposes that appearance-related pressures from media and other social sources contribute to body-related cognitions through appearance-based social comparison and internalization of appearance ideals, which may shape health-related attitudes and behaviors. Emerging evidence from pregnancy samples supports these pathways, suggesting indirect links between sociocultural pressures and body dissatisfaction via comparison and internalization processes^15^. Because appearance-based comparison and ideal internalization were not directly measured in the present study, TIM is used here as a conceptual lens to interpret associations between perceived media portrayals, body esteem, and health-related behaviors rather than to test a full mediational pathway.

The present study aims to identify clinical and psychosocial correlates of health behaviors among pregnant women, with a particular focus on perceptions of media portrayals of pregnancy and body image. It represents an attempt to fill the research gap by integrating clinical, psychological and media data in the analysis of perinatal health behaviors. We hypothesized that: (H1) clinical factors (gestational age and comorbidities) would be associated with health-promoting behaviors; (H2) more negative perceived media portrayals of pregnancy would be associated with lower levels of health-promoting behaviors, whereas more positive portrayals would be associated with higher levels; and (H3) higher body esteem (BES subscales) would be positively associated with health-promoting behaviors. Accordingly, we investigated how clinical factors, body esteem, and perceived media portrayals of pregnancy are associated with health-promoting behaviors.

## Results

### Sociodemographic and clinical characteristics

The mean age of the respondents was 30.65 years (SD = 4.90). Mean height was 166.1 cm (SD = 6.6) and mean pre-pregnancy weight was 66.3 kg (SD = 14.8). Most of the respondents lived in a provincial city (59.0%), had a university degree (62.3%), were in stable relationships (79.4%), and rated their family’s financial situation as average (79.8%). Most respondents were experiencing their first pregnancy (47.4%), reported a low-risk pregnancy (73.2%), and reported no comorbidities (59.2%); 49.9% of respondents reported being satisfied with their health. A sense of attractiveness during pregnancy was reported by 64.3% of respondents, and 49.9% accepted the changes taking place in their bodies. Concerns about appearance postpartum were reported by 45.4% of respondents, and 44.2% of respondents said that media portrayals of pregnancy are unrealistic (Table 1).

**Table 1:** Sociodemographic and clinical characteristics of respondents.

Characteristics		*n*	%
Mean age/years (M, SD; range)		30.65 (4.90); 18–45	
Height/cm (M, SD; range)		166.1 (6.6); 138–187	
Pre-pregnancy weight/kg (M, SD; range)		66.3 (14.8); 42–172	
Place of residence	Provincial city	543	59.0
	Other city	140	15.2
	Rural area	238	25.8
Education	Primary	96	10.4
	Secondary	251	27.3
	Higher	574	62.3
Marital status	Single	190	20.6
	Married/in a relationship	731	79.4
Perceived Family Wealth	Rich	160	17.4
	Average	735	79.8
	Poor	26	2.8
Number of pregnancies	1	437	47.4
	2	298	32.4
	3 and more	186	20.2
Course of pregnancy	Low-risk pregnancy	674	73.2
	High-risk pregnancy	247	26.8
Occurrence of comorbidities	No	545	59.2
	Yes	376	40.8
Self-assessment of health status	Positive	460	49.9
	Neither good nor bad	358	38.9
	Negative	103	11.2
Sense of attractiveness	Yes	592	64.3
	No	329	35.7
Acceptance of changes occurring during pregnancy	I accept my body	460	49.9
	I do not pay attention to it	379	41.1
	I do not accept my body	82	8.9
Concern about appearance after childbirth	No	503	54.6
	Yes	418	45.4
Image of pregnant women in the media	Positive	339	36.8
	Unrealistic	407	44.2
	Negative	93	10.1
	Media underrepresentation	82	8.9

M-mean; SD-standard deviation.

### Descriptive statistics for key psychological variables

The mean score for positive health behaviors (PHBS) was 50.50 (SD = 14.98). The mean scores for body esteem (BES) subscales were as follows: sexual attractiveness – 44.11 (7.90), weight concern – 32.44 (7.32), and physical condition – 29.20 (6.71).

### Hierarchical regression analysis of predictors of positive health behaviors

Four models were tested in a hierarchical regression analysis in which blocks of variables were introduced sequentially: clinical-health (Model 1), psychological (Model 2), perceptions of media portrayals of pregnancy (Model 3), and components of the BES scale (Model 4). Each successive step of the analysis significantly improved the fit of the model, as confirmed by the values of the F statistic and the increase in the R² coefficient of determination. The final model explained 87% of the variance in health behaviors among pregnant women in this sample (R² = 0.87, F = 433.54, *p* < 0.001) – Table 2.

**Table 2:** Analysis of variance (ANOVA) comparing the fit of successive hierarchical regression models for predictors of health behavior.

Model	Regression sum of squares	Mean square regression	F	*p*
Model 1	2690.57	672.64	3.537	0.007
Model 2	4702.23	671.75	3.562	0.001
Model 3	14543.18	1322.11	7.403	< 0.001
Model 4	153642.01	10974.43	433.539	< 0.001

Table 3 shows the fit of successive hierarchical regression models for positive health behaviors (PHBS). At each stage, the introduction of new blocks of predictors was associated with a statistically significant increase in explained variance (*p* < 0.01 for all models). The highest level of fit was achieved in the final model (Model 4), which included BES subscales. Detailed results are presented in the table.

**Table 3:** Model fit indices for hierarchical regression predicting positive health behaviors among pregnant women.

Model	*R*	*R*²	Adjusted *R*²	F	*p*
Model 1	0.123	0.015	0.011	3.537	0.007
Model 2	0.163	0.027	0.019	3.562	< 0.001
Model 3	0.287	0.082	0.071	7.403	< 0.001
Model 4	0.933	0.87	0.868	433.539	< 0.001

### Predictive role of clinical, psychological and media-related variables

The results of the final hierarchical regression model indicate that a combination of clinical factors, body self-esteem components, and perception of media messages are associated with health behaviors among pregnant women. In the first stage, significant effects were shown by week of pregnancy (B = -0.161; *p* < 0.001), with lower levels of health behaviors observed at later gestational weeks, and the presence of comorbidities (B = 2.871; *p* < 0.001), which was associated with a higher frequency of positive health behaviors. In the second stage, the body perception variables did not reach the level of statistical significance (*p* > 0.05). In the third stage, using “unrealistic” portrayals as the reference category, perceiving portrayals as negative was significantly associated with lower levels of health behaviors (B = − 2.938; *p* < 0.001), whereas perceiving portrayals as positive (B = 1.037; *p* = 0.015) and a lack of representation (“**media underrepresentation**”; B = 2.120; *p* = 0.005) were associated with higher levels. In the final model, all BES subscales were significant predictors: sexual attractiveness (B = 0.259; *p* < 0.001), weight concern (B = 0.563; *p* < 0.001), and physical condition score (B = 0.812; *p* < 0.001). Each of these factors was positively associated with positive health behaviors among respondents – Table 4.

**Table 4:** Hierarchical linear regression model for predictors of positive health behaviors among respondents.

Predictor	B	t	*p*	95% CI LL	95% CI UL	Tolerance	VIF
(Constant)	-19.821	8.585	< 0.001	-24.352	-15.29		
STEP 1. Clinical and health variables							
Week of pregnancy	-0.161	-3.983	< 0.001	-0.241	-0.082	0.634	1.58
Course of pregnancy	0.003	0.007	0.995	-0.749	0.754	0.879	1.14
Occurrence of comorbidities	2.871	7.67	< 0.001	2.137	3.606	0.934	1.07
Self-assessment of health status	0.465	1.605	0.109	-0.104	1.034	0.823	1.21
STEP 2. Perception of body and image during pregnancy							
Sense of attractiveness	-0.654	-1.86	0.063	-1.345	0.036	0.769	1.30
Acceptance of change occurring during pregnancy (higher score = lower acceptance)	0.165	0.511	0.609	-0.47	0.8	0.719	1.39
Concern about appearance after childbirth	-0.718	-1.923	0.055	-1.451	0.015	0.908	1.10
STEP 3. Perceived media portrayals of pregnancy							
Positive vs. Unrealistic	1.037	2.435	0.015	0.201	1.873	0.768	1.30
Negative vs. Unrealistic	-2.938	-4.035	< 0.001	-4.366	-1.509	0.807	1.24
Media underrepresentation vs. Unrealistic	2.120	2.845	0.005	0.658	3.582	0.821	1.22
STEP 4. Body Self-Esteem Components (BES)							
BES Sexual Attractiveness	0.259	10.136	< 0.001	0.209	0.309	0.304	3.29
BES Weight Concern	0.563	9.787	< 0.001	0.5	0.626	0.405	2.47
BES Physical Condition	0.812	11.319	< 0.001	0.733	0.891	0.348	2.87

CI – Confidence interval; LL – Lower limit; UL – Upper limit; VIF – Variance inflation factor.

Media portrayals were coded with “Unrealistic” as the reference category. Acceptance of body changes during pregnancy was modeled as an ordinal predictor coded 1–3 (1 = accept body changes, 2 = do not pay attention, 3 = do not accept body changes); higher values indicate lower acceptance.

## Discussion

This study showed that health-promoting behaviors during pregnancy are associated with a combination of clinical factors, body esteem dimensions, and perceived media portrayals of pregnancy. Our findings can be interpreted within sociocultural frameworks, particularly the Tripartite Influence Model, which conceptualizes body image as a dynamic process shaped by sociocultural pressures and their internalization via appearance-based social comparison and ideal internalization. Empirical work in pregnant samples supports these pathways, showing that media and other sociocultural pressures (including family and significant others) are linked to body dissatisfaction indirectly via appearance-based comparison and thin-ideal internalization^15^. While the model also highlights peer, family, and partner influences, the present study assessed the sociocultural context primarily through perceived media portrayals of pregnancy. The high proportion of explained variance in the final model should be interpreted cautiously, as it may partly reflect conceptual overlap and shared-method variance among psychosocial self-report predictors and health-promoting behaviors. Collinearity diagnostics (tolerance/VIF) suggested no problematic multicollinearity. To reflect the hypothesized ordering of determinants, predictors were entered hierarchically from clinical background factors to psychological variables, perceived media portrayals, and body esteem dimensions. This approach aligns with a biopsychosocial perspective and previous empirical work^16^. Body esteem dimensions may be viewed as intrapersonal resources related to self-evaluation and self-regulation that can support engagement in health-promoting behaviors^6^.

Among the clinical-health variables, gestational age was associated with lower levels of health-promoting behaviors. This pattern is consistent with reports of reduced physical activity and less consistent adherence to dietary and health recommendations in late pregnancy^5,17^ and may reflect increasing fatigue, reduced mobility, and a higher burden of somatic complaints^1,18^.

Comorbidities were associated with higher levels of health-promoting behaviors. This may reflect more frequent medical monitoring and more intensive guidance among individuals managing chronic conditions, which can facilitate engagement in recommended practices. Similar patterns have been reported in pregnancies complicated by gestational diabetes or hypertensive disorders^19–21^. This group may also benefit from tailored educational support within prenatal care (e.g., nurse- and midwife-led counselling) to help sustain adaptive behaviors across pregnancy. Notably, subjective self-rated health and perceived course of pregnancy were not associated with health behaviors, suggesting that perceived well-being does not always translate into lifestyle-related action^1^. Overall, these findings highlight meaningful clinical differences that should be considered when designing preventive and educational interventions.

In our sample, brief appearance-focused appraisals (e.g., attractiveness, acceptance of changes, concerns about appearance after childbirth) were not closely related to health-promoting behaviors. This may indicate that surface-level appearance evaluations during pregnancy are less behaviorally relevant than broader body esteem, which may reflect more stable self-evaluations linked to self-regulation. One plausible explanation is that pregnancy-related bodily changes are often perceived as temporary.

Modern media, especially social media, may influence pregnant women’s health beliefs and behaviors^22,23^. In our study, negative media portrayals of pregnancy were associated with lower levels of health-promoting behaviors, consistent with reports linking exposure to unrealistic content with lower body-related self-evaluation and greater perinatal distress^24,25^. This literature largely comes from European and North American (Global North) samples. Our findings extend it to a Polish (Central/Eastern Europe) context. Appearance-focused messages may foster body dissatisfaction and may undermine health-promoting attitudes, potentially via appearance-based comparison and internalization of unrealistic ideals^23,26,27^. Excessive social media use has been associated with greater social comparison, depressive symptoms, anxiety, and body image disturbance^28,29^. In contrast to negatively perceived messages, positive representations of pregnant women that promote the image of an active, strong, and caring woman were associated with higher levels of health-promoting behaviors. Such content can provide psychological support, enhance a sense of empowerment, motivation to take care of one’s health, and foster a positive perception of one’s own body^30^. Drawing on social learning theory, exposure to attainable and supportive role models may increase the likelihood of adopting similar attitudes and behaviors^31^. Public campaigns such as *Start4Life* (UK) and *Pregnancy*,* Birth*,* and Baby* (Australia) demonstrate this, combining health education with realistic and responsible messages tailored to pregnant women’s needs^32,33^. These examples suggest how health systems, public institutions, and digital environments can align to support perinatal health.

Interestingly, both positive content and “media underrepresentation” (lack of representation) were associated with more frequent health-promoting behaviors. While the potential benefits of supportive content are described in the literature, the association between lack of representation and health-promoting behaviors should be interpreted cautiously and may reflect heterogeneous processes^30,31^. For some women, limited reliance on media role models may coincide with access to reliable information and professional support through prenatal care^31,34^. From this perspective, “media underrepresentation” may function as a neutral background that is supplemented by trusted sources such as nurses, midwives, or prenatal educators. At the same time, the absence of appearance-focused content may reduce opportunities for unrealistic comparison, which could support more grounded body acceptance and engagement in health-promoting practices^35^. These interpretations are tentative, as the proposed mediating processes (e.g., internalization of ideals and appearance-based comparison) were not directly measured in this study.

In the final model, all three BES scale subscales – sexual attractiveness, weight concern, and physical condition, were significant predictors of health-promoting behaviors among pregnant women. In line with sociocultural accounts (e.g., internalization and appearance-based comparison), more functional and relational facets of body esteem (physical condition and sexual attractiveness) may be more proximally linked to motivation and self-regulation for health-promoting practices than more surface-level appearance appraisals. The strongest association was observed for physical condition, suggesting that perceived fitness and bodily functionality may be particularly salient correlates of engagement in health-promoting behaviors. Notably, despite its predictive strength, this subscale had the lowest mean score, which may reflect physiological constraints of pregnancy such as fatigue, pain, and reduced mobility^36^. More positive body-related self-evaluation, including not only appearance but also functionality, has been associated with greater engagement in physical activity, healthier dietary practices, and adherence to medical recommendations^35,37^. Interventions tailored to pregnant women’s capabilities may support perceived physical condition, while regular physical activity may enhance self-efficacy and a sense of agency regarding one’s own and the baby’s health^38,39^.

Weight concern was a positive predictor of health-promoting behaviors, suggesting that moderate concern may serve a motivational and adaptive function. It may be linked to adaptive choices such as physical activity and a balanced diet, consistent with recommendations for gestational weight gain^6,40,41^. Given the widespread use of the Institute of Medicine (IOM) gestational weight gain guidance, weight concern in pregnancy may partly reflect weight-normative pressures and stigma, which in the broader literature have been linked to lower body image and eating-related difficulties^42^. However, pre-pregnancy BMI and prior health behaviors may shape weight-related beliefs during pregnancy, influencing both the desire for control and perceived control over weight change^43^. At the same time, pregnancy can be a period of increased vulnerability to body dissatisfaction, particularly among women with elevated anxiety or depressive symptoms. In such cases, negative body-related self-evaluation may be associated with compensatory behaviors such as restrictive eating or excessive exercise^44,45^. Therefore, weight concern may be protective only within certain bounds and may become maladaptive when intensified by unrealistic ideals or stigmatizing messages, potentially contributing to insufficient gestational weight gain or chronic appearance-related stress^40,46^. This complexity highlights the need for interventions that combine health education with psychological support and that address both sociocultural pressures and individual vulnerability factors. Health promotion in pregnancy should prioritize well-being and evidence-based guidance rather than appearance-focused norms.

Sexual attractiveness was also associated with higher levels of health-promoting behaviors among pregnant women. This subscale had the highest mean scores, suggesting relatively positive sexual self-perceptions during pregnancy. A more positive sense of sexual attractiveness may support self-care behaviors (e.g., adherence to medical recommendations, healthier eating, and physical activity), potentially through relationship and psychosocial pathways. Consistent with this view, partner-related research links perceived attractiveness with relationship quality and sexual satisfaction, and supportive partner responses may buffer anxieties about bodily changes^47,48^. Conversely, lower perceived attractiveness has been associated with lower sexual functioning, and persistent taboos around sexuality during pregnancy may contribute to concerns about safety and bodily changes^49,50^.

Overall, BES dimensions, particularly perceived physical condition, were most strongly associated with health-promoting behaviors, while weight concern appeared potentially adaptive only within certain bounds. Together, the findings underscore the value of addressing body-related self-evaluations and sociocultural influences in prenatal care. This integrated view aligns with biopsychosocial models emphasizing interplay of biological, psychological, and sociocultural factors in health behavior^51^.

Strengths of this study include its extensive geographic scope; recruiting respondents from several major provinces increases sample representativeness and supports generalizability. The research design encompassed a wide range of variables, not only demographic and clinical, but also psychosocial aspects and perceptions of social media influence. This holistic approach supports a better understanding of the complex determinants of health behavior. The use of standardized psychometric tools ensured accurate measurement and enhanced data reliability. In addition, multivariate regression allowed for the simultaneous consideration of multiple predictors and identification of key determinants, providing directions for preventive, educational, and policy-level interventions.

However, the study has certain limitations. Its cross-sectional design does not allow for conclusions about causal relationships between psychosocial variables and health behaviors. Establishing the direction of these relationships would require longitudinal research. The use of self-report questionnaires may have introduced bias due to social desirability, especially in responses related to body image and health behaviors. The relatively high proportion of explained variance should also be interpreted with caution, as it may partly reflect conceptual overlap and shared-method variance among psychosocial self-report measures and health behaviors. Additionally, the assessment of media influence was based on participants’ subjective perceptions rather than objective content analysis, which may have affected the precision of these estimates. Although the sample was geographically diverse, it was not based on a nationally random sampling strategy; therefore, the generalizability of the findings should be interpreted with caution. We did not assess gender identity beyond participants’ self-identification as women. Therefore, the findings may not generalize to all individuals who can become pregnant (e.g., transgender men, non-binary and gender-diverse individuals). In addition, much of the available literature we cite is based on samples of cisgender women, which may further limit the generalizability of gender-related inferences. Despite these limitations, the findings offer valuable insights into the psychosocial factors influencing health behaviors during pregnancy and can inform future research that tests mechanisms more directly.

These findings have several practical implications for prenatal care and health education. The psychosocial factors highlighted in the study, including body acceptance and interpretation of media messages, can be considered when evaluating well-being during pregnancy. In clinical settings, it may be advisable to implement interventions that promote a positive body image and strengthen a sense of self-efficacy and empowerment, particularly within prenatal counselling for women experiencing appearance-related anxiety or low self-esteem.

Educational programs targeting pregnant women may promote a realistic, inclusive view of pregnancy that takes into account both the natural physical changes and emotional challenges of this period. Emphasizing that bodily diversity during pregnancy is normal and acceptable can help reduce psychological tension and promote the adoption of health-promoting behaviors. At the same time, the media, as a major source of social influence, could shift away from unrealistic, one-dimensional portrayals of pregnancy and instead present more authentic and diverse representations. Encouraging realism and inclusivity in media content may help minimize harmful social comparisons, enhance body acceptance, and support psychological well-being during pregnancy; such efforts may reduce appearance-based comparison and internalization of unrealistic ideals.

Health content creators, including parenting influencers and social media platforms, may play an important role in shaping pregnant women’s health-related attitudes and habits. A prerequisite for a positive influence is message credibility, alignment with evidence-based guidance, and avoidance of unrealistic aesthetic standards. Promoting authentic, diverse representations of pregnant women may enhance positive self-esteem and foster more informed health decision-making.

At the systemic level, incorporating media literacy into prenatal education programs, particularly those delivered by nurses and other healthcare educators may be justified, given the growing impact of social media as a source of health-related information. This strategy should aim not only to enhance the quality and accessibility of reliable digital resources but also to strengthen individuals’ ability to critically evaluate media content.

## Methods

### Study design

The study included 921 pregnant women receiving care at medical facilities in six provinces in Poland (Central/Eastern Europe): Lubelskie, Mazowieckie, Podkarpackie, Podlaskie, Zachodniopomorskie, and Wielkopolskie. The study was carried out between December 2023 and July 2024. All participants were informed that participation was voluntary and anonymous. They were informed that they could withdraw at any time without penalty, and that any data collected would be used only for research purposes. Participants meeting the following criteria were eligible to participate in the study: age of majority (≥ 18 years), native Polish language, singleton pregnancy, and voluntary consent to participate. Individuals under 18 years of age, those with multiple pregnancies, or those whose condition required hospitalization during the study were excluded. Gender identity was not assessed beyond participants’ self-identification as women. Data were collected using a self-administered questionnaire during routine prenatal visits at participating facilities. The questionnaire was distributed and completed on-site in a private setting, ensuring anonymity and comfort for participants. The study was approved by the Bioethics Committee of the Karol Marcinkowski Medical University in Poznań, Poland (Resolution No. 931/23, approved on 7 December 2023). All procedures were performed in accordance with the ethical standards of the institutional research committee, the principles of the Declaration of Helsinki, and relevant national guidelines and regulations. Written informed consent was obtained from all participants prior to their inclusion in the study.

### Data collection

The study used a cross-sectional survey design. The survey tools were: Positive Health Behavior Scale (PHBS), Body Esteem Scale (BES) and a structured self-administered questionnaire containing items used to characterize the participants. The structured questionnaire included items on demographic variables (age, place of residence, marital status, education, perceived material situation), clinical-health variables (week of pregnancy, course of pregnancy, presence of comorbidities, self-assessment of health), perception of one’s own body and image during pregnancy (sense of attractiveness during pregnancy, acceptance of changes during pregnancy, concerns about appearance after delivery). In addition, we assessed perceptions of media portrayals of pregnant women and participants’ subjective evaluation of their own body.

The Positive Health Behavior Scale (PHBS) by Hildt-Ciupinska measures the frequency with which individuals engage in health-promoting activities in five areas of functioning: nutrition, body care, personal safety, psychosocial health and physical activity. Respondents indicated how often they engaged in each behavior using a four-point response scale: from “almost never” (0 points) to “almost always” (3 points). The total score is the sum of the points and is an indicator of the overall level of health-promoting behaviors. The tool has good psychometric reliability (Cronbach’s α = 0.81)^52^. In the present sample, internal consistency was high (Cronbach’s α = 0.92).

The Body Esteem Scale (BES) by Franzoi and Shields in the Polish adaptation by Lipowska and Lipowski measures three dimensions of self-assessment of the body: sexual attractiveness, weight concern, and assessment of physical condition. It consists of 35 items rated on a five-point Likert scale, where higher values indicate more positive attitudes toward a particular aspect of appearance. The BES has high reliability: Cronbach’s alpha is 0.87 for the sexual attractiveness subscale, 0.90 for the weight concern subscale, and 0.87 for the physical condition rating^53,54^. In the present sample, internal consistency was high for the overall BES (Cronbach’s α = 0.95) and for the subscales: Sexual Attractiveness (α = 0.88), Weight Concern (α = 0.91), and Physical Condition (α = 0.87).

### Statistical analysis

Statistical analyses were performed using IBM SPSS Statistics (version 29). Categorical variables were presented as counts (N) and percentages (%), and continuous variables as means (M) and standard deviations (SD). Missing data were minimal (≤ 0.16% for key variables). Little’s MCAR test for key continuous variables was non-significant (*p* = 0.43); therefore, regression analyses were conducted using complete-case (listwise) deletion. Hierarchical linear regression, including four blocks of variables, was used to identify factors associated with positive health behaviors. Model fit was assessed using changes in R² and F-tests (ANOVA) across regression steps. The hierarchical entry of predictor blocks was conceptually guided by a biopsychosocial perspective, progressing from clinical factors to psychological variables, perceived media portrayals, and body esteem dimensions. For each predictor, we report unstandardized regression coefficients (B), 95% confidence intervals (CIs), and p-values. Collinearity was evaluated using tolerance and variance inflation factors (VIF). The significance level was taken as *p* < 0.05.

## Conclusion

A complex interplay of clinical, psychological, and sociocultural factors was associated with health-promoting behaviors during pregnancy. Understanding these behaviors may benefit from an integrated perspective that considers these dimensions collectively. In this sample, more advanced gestational age was associated with lower levels of health-promoting behaviors, which may reflect physical limitations and reduced activity in later pregnancy. In contrast, the presence of comorbidities was associated with higher levels of such behaviors, possibly reflecting increased health monitoring and greater motivation to adopt preventive practices.

Perceived media portrayals of pregnancy were also associated with health-promoting behaviors: lack of representation (“media underrepresentation”) and positive portrayals were linked to higher engagement, whereas negative portrayals were linked to lower engagement.

The strongest associations in the final model were observed for Body Esteem Scale dimensions. A stronger sense of sexual attractiveness, weight concern, and a more positive perception of physical condition were all associated with more frequent engagement in health-promoting behaviors. These findings suggest that positive body self-evaluation may function as a relevant psychological resource during pregnancy. They also support the value of a holistic approach to health promotion during pregnancy that combines medical care with psychological support and attention to the sociocultural context of health-related decisions. The findings may help inform the development of educational and preventive programs that promote constructive media messaging and support a healthier body image during pregnancy.

Given the cross-sectional design, these findings should be interpreted as associations rather than causal relationships, and longitudinal studies are needed to clarify directionality and mechanisms.

## Supplementary Information

Below is the link to the electronic supplementary material.

Supplementary Material 1

Supplementary Material 2

## Data Availability

The datasets generated and/or analyzed during the current study are not publicly available due to sensitivity and privacy concerns, but are available from the corresponding author on reasonable request.
