# Supplementary material for: Body image and social media as predictors of pregnancy health behaviors
Source: Sci Rep. 2026 Mar 5;16:12175. doi: 10.1038/s41598-026-43123-5 (PMC13076754; doi:10.1038/s41598-026-43123-5)
Supplement: Supplementary file 1 — Supplementary Material 1 [file 41598_2026_43123_MOESM1_ESM.pdf]

QUESTIONNAIRE

Dear Participant,
You are kindly invited to participate in a study aimed at identifying factors influencing health behaviors and body self-esteem among pregnant women.
Please complete the following questionnaire and provide sincere and comprehensive answers to all questions. Read each question carefully together with the response options and mark your chosen answer with an "X" in the appropriate box or fill in the blank space where indicated. Please note that the questionnaire is anonymous and the collected data will be used solely for scientific purposes.

Thank you very much for your time.

1. How old are you? ……………………..
2. Place of residence:
   a. Provincial city
   b. Other city
   c. Rural area
3. Marital status:
   a. Single
   b. Married / in a relationship
   c. Not in a relationship
4. Education:
   a. Primary / vocational
   b. Secondary
   c. Higher
5. How do you assess your family's financial situation (Perceived Family Wealth, PWF)?
   a. Rich
   b. Average
   c. Poor
6. What is your height (in cm)? ……………………..
7. What was your body weight before pregnancy (in kg)? ……………………..
8. What is your current body weight (in kg)? ……………………..
9. How many pregnancies have you had, including the current one? ……………………..
10. What week of pregnancy are you currently in? ……………………..
11. Do you have children?
    a. Yes
    b. No
12. Is your current pregnancy progressing normally, without complications?
    a. Yes
    b. No
13. How do you assess your health status?
    a. Positive
    b. Neither good nor bad
    c. Negative
14. Do you suffer from any conditions such as hypertension, diabetes, thyroid disease, heart disease, allergies, etc.?
    a. No
    b. Yes, if yes, please specify: ……………………..
15. Do you feel attractive during pregnancy?
    a. Yes
    b. No
16. How do you feel about the changes occurring in your body during pregnancy?
    a. I accept my body
    b. I do not pay attention to it
    c. I do not accept my body
17. Are you concerned about excessive weight gain during pregnancy?
    a. No
    b. Yes
18. Are you concerned about your appearance after childbirth?
    a. No
    b. Yes
19. Do you use social media such as Facebook, Instagram, or TikTok?
    a. Yes
    b. No

20. In your opinion, how do the media portray pregnant women?
    a. Pregnant women are portrayed as well-groomed, independent, and caring about their appearance
    b. The media create an unrealistic, idealized image of pregnant women
    c. The media present a negative image of pregnant women
    d. There is "media underrepresentation" of pregnant women
21. Does the image of pregnant women presented in the media influence how you perceive your own body?
    a. No
    b. Yes
    c. I have no opinion on this matter

Body Esteem Scale (BES)

Below are various aspects related to your body. Please rate each item by marking "X" next to one of the five response options (from 1 to 5).

**Item**	I have strong negative feelings	I have moderate negative feelings	I have no feelings	I have moderate positive feelings	I have strong positive feelings
1. Body scent	1	2	3	4	5
2. Appetite	1	2	3	4	5
3. Nose	1	2	3	4	5
4. Physical stamina	1	2	3	4	5
5. Reflexes	1	2	3	4	5
6. Lips	1	2	3	4	5
7. Muscular strength	1	2	3	4	5
8. Waist	1	2	3	4	5
9. Energy level	1	2	3	4	5
10. Thighs	1	2	3	4	5
11. Ears	1	2	3	4	5
12. Arms	1	2	3	4	5
13. Chin	1	2	3	4	5
14. Body build	1	2	3	4	5
15. Physical coordination	1	2	3	4	5
16. Buttocks	1	2	3	4	5
17. Agility	1	2	3	4	5
18. Shoulder width	1	2	3	4	5
19. Hands	1	2	3	4	5
20. Breasts / chest	1	2	3	4	5
21. Eyes	1	2	3	4	5
22. Cheeks / cheekbones	1	2	3	4	5
23. Hips	1	2	3	4	5
24. Legs	1	2	3	4	5
25. Figure	1	2	3	4	5
26. Sex drive	1	2	3	4	5
27. Feet	1	2	3	4	5
28. Sex organs	1	2	3	4	5
29. Stomach	1	2	3	4	5
30. Health	1	2	3	4	5
31. Sexual activities	1	2	3	4	5
32. Body hair	1	2	3	4	5
33. Physical condition	1	2	3	4	5
34. Face	1	2	3	4	5
35. Weight	1	2	3	4	5

Positive Health Behaviors Scale

Please read each of the following statements carefully and consider how often you behave in the described way. Please place an "X" in one box in each row.

	Always	Very often	Someti mes	Rarely
**I. Nutrition subscale**				
1. I eat at least 3 meals a day at similar times.	☐	☐	☐	☐
2. I eat breakfast at home every morning (i.e., something more than a glass of milk, tea, or another beverage).	☐	☐	☐	☐
3. I eat fruit at least once a day.	☐	☐	☐	☐
4. I eat vegetables at least once a day.	☐	☐	☐	☐
5. I drink at least 2 glasses of milk, kefir, or yogurt every day.	☐	☐	☐	☐
6. I limit eating sweets.	☐	☐	☐	☐
7. I avoid snacking between meals (e.g., between lunch and afternoon snack, or between afternoon snack and dinner).	☐	☐	☐	☐
**II. Body care subscale**				
8. I dress appropriately for the weather (i.e., I do not expose myself to cold and I do not overheat).	☐	☐	☐	☐
9. I avoid excessive sun exposure (e.g., I use sunscreen, cover my head, and avoid sunbathing between 10 a.m. and 2 p.m.).	☐	☐	☐	☐
10. I brush my teeth at least twice a day.	☐	☐	☐	☐
11. I visit the dentist for check-ups every 6 months.	☐	☐	☐	☐
12. I undergo cervical cytology screening once a year.	☐	☐	☐	☐
13. I perform breast self-examination once a month.	☐	☐	☐	☐
**III. Safety behavior subscale**				
14. I fasten my seatbelt when traveling by car.	☐	☐	☐	☐
15. I wear a helmet while riding a bicycle (if you do not ride a bicycle, please leave this row blank).	☐	☐	☐	☐
16. I follow traffic rules when walking, cycling, or driving a car.	☐	☐	☐	☐
17. I behave safely near water (e.g., I swim only in supervised areas, do not dive headfirst, and wear a life jacket when boating or kayaking).	☐	☐	☐	☐
18. I follow safety rules when using electrical devices, machinery, or chemical substances.	☐	☐	☐	☐
**IV. Psychosocial health subscale**				
19. I sleep at least 7–8 hours at night.	☐	☐	☐	☐
20. I go to bed at the same time each night.	☐	☐	☐	☐
21. I spend at least 20–30 minutes a day relaxing/resting (e.g., I do relaxation exercises or do things I enjoy).	☐	☐	☐	☐
22. I cope well with excessive stress (tension).	☐	☐	☐	☐
23. I think positively about myself and the world.	☐	☐	☐	☐
24. I ask other people for help in situations that are difficult for me (e.g., family, friends).	☐	☐	☐	☐
25. I spend time with acquaintances/friends at least once a month.	☐	☐	☐	☐
**V. Physical activity subscale**				
26. I spend at least 30 minutes every day on activities involving moderate or vigorous physical effort (e.g., jogging, brisk walking, sports, gardening, or farm work).	☐	☐	☐	☐
27. I participate in organized physical activity classes or training sessions at least once a week.	☐	☐	☐	☐
28. I increase the amount of movement and physical activity in daily life (e.g., I walk instead of going by car, bus, or tram, and I take the stairs instead of the elevator).	☐	☐	☐	☐
29. I watch television no longer than 2–3 hours a day.	☐	☐	☐	☐

Thank you very much for participating in the survey.
